# The Late Mr. Durance George

**Published:** 1852-01

**Authors:** 


*The late Mr. Durance George.*—It is with feelings of regret we announce the premature death of this distinguished and accomplished dentist, at his residence, Old Burlington Street, London.

Mr. George was nephew to the veteran Cartwright, and prosecuted his studies at the London University as a surgeon—upon the anticipated retirement of his uncle, he abandoned general surgery and commenced his studies by familiarizing himself with the labors of the work bench, under the able tuition of the late Mr. Sherwin. After devoting some time to this branch, he commenced his career as an assistant to Mr. Cartwright, and upon his retirement he succeeded to the practice. Even at this early period he began to be known and appreciated in dental practice, for in the autumn of the year 1843, he was requested to deliver a course of lectures on physiology and diseases of the teeth, at the Theatre adjoining St. George's Hospital; the following year the University College appointed him to the professorship of dental surgery. The lectures were distinct, comprehensive and well arranged—embodying all the objects usually contained in such a course—not confining himself to the mechanical, surgical and operative treatment of the dental organs; he arranged a mass of information on their pathological relations, and morbid anatomy, illustrating important points by means of a collection of diagrams, drawings and models of the best kind. As a lecturer, Mr. George's manner was unembarrassed, his language fluent and well selected, his arrangement clear and comprehensive, while he introduced any novel or important facts by modestly avowing that the opinions he advanced were not his alone, but supported by the sanction of men much older, and experienced in the profession. As a physiologist, Mr. George stood deservedly high, at the same time, he displayed great mechanical ingenuity in the practical treatment of the dental organs.

As a private man, his manners are said to have been courteous and gentlemanly—whilst, with the liberality accompanying real merit, he imparted the results of his professional experience to his less fortunate brethren, without hesitation or professional jealousy, and thus secured to himself a large circle of professional friends. Mr. George had been ailing for the last two or three years, but his most intimate friends did not anticipate any serious danger, but considered by relaxation from professional fatigues he would ultimately recover—it was only within a month or so previous to his death, they were aware of his dangerous condition—and as the time approached for delivering his introductory lecture at the college, it was postponed in consequence of his indisposition. After a few days, he delivered his lecture with evident signs of physical and mental sufferings, and apologized to his class for the previous dissappointment, and his present want of physical power to do justice to the subject—but announced to his pupils the day and hour he should commence the course—upon the pupils assembling, they learnt the alarming state their professor was in, and no hopes entertained of his ultimate recovery. On the 20th of November, four days after his introductory lecture, this distinguished and accomplished dental practitioner ended his earthly career at the early age of thirty-six—in the prime of life, with his intellectual faculties full of vigor—but the physical, worn and injured by over exertion and devoted attachment to his profession.

